# South African midwife specialists’ experiences in the utilisation of their knowledge and skills

**DOI:** 10.4102/hsag.v29i0.2444

**Published:** 2024-03-18

**Authors:** Kagiso P. Tukisi, Zelda Janse van Rensburg, Wanda Jacobs

**Affiliations:** 1 Department of Nursing, School of Health Care Sciences, Sefako Makgatho Health Sciences University, Pretoria, South Africa; 2 Department of Nursing, Faculty of Health Sciences, University of Johannesburg, Johannesburg, South Africa

**Keywords:** midwife specialists and neonatal nurse, specialist, knowledge and skills, scope of practice, litigations

## Abstract

**Background:**

Midwifery specialisation was introduced in 1993 as a response to escalating maternal and neonatal mortalities and shortage of physicians in rural parts of South Africa. Basic midwives enrolled into a postgraduate midwifery qualification to extend their knowledge and skills which enabled them to manage complicated obstetric conditions. The postgraduate midwifery qualification rendered them midwife specialists upon completion of the course. Yet, MS remain underutilised in clinical facilities due to limiting practice regulations and fear of medico-legal litigations, leading to forfeiture of skills.

**Aim:**

The study aimed to explore and describe midwife specialist’ experiences of optimal utilisation of their knowledge and skills in public health facilities in South Africa.

**Setting:**

Public health facilities based in seven provinces in South Africa where MS were employed, formed part of the research setting.

**Methods:**

A qualitative, descriptive and explorative research design was followed using phenomenological approach. Sixteen purposefully sampled midwife specialists participated in four focus group interviews. Data were analysed using Collaizi’s descriptive method.

**Findings:**

Three themes, each with categories, were derived from the data. Research results confirmed midwife specialist’ limited utilisation of knowledge and skills in public facilities. This was associated with the existing practice regulations, restricting midwife specialists to basic midwifery roles.

**Conclusion:**

The lack of practice regulations, particularly for midwife specialists hinders optimal utilisation of their knowledge and skills in the public health facilities.

**Contribution:**

This study highlighted midwife specialist’ barriers in optimally embracing their expert knowledge and skills. Barriers may guide formulation of strategies to facilitate midwife specialist’ knowledge and skills utilisation.

## Introduction

Midwife specialists are registered professional nurses who have completed the specialist diploma in midwifery. Upon completion of the diploma, they are registered as midwife specialists with the South African Nursing Council (SANC 2014). The midwife specialist diploma training programme (previously referred to as the ‘post basic midwifery and neonatal nursing diploma’) was first introduced in South Africa in 1993 (Sellers et al. 2018). The training programme was made available for basic midwives to train as midwife specialists, referred to as ‘midwife specialists’. Being qualified as a midwife specialist had the added benefit to address the maternal and child mortality in South Africa, and meet the requirements of the re-engineered primary health care system amid staff shortages (Maree, Yazbek & Leech 2018). It was proposed that midwife specialists would have the knowledge and skills to curb the shortage of physicians in rural parts of South Africa (Sewnunan & Puckree, 2022).

The midwife specialist qualification signifies a specialised field in nursing, comprising expanded and expert roles, knowledge, skills and competencies of the midwife to improve maternal health, reproductive and genetic counselling and neonatal health (SANC 2014). South Africa is not the only country that pursued expansion of knowledge and skills of midwives; countries such as Liberia and Uganda train their midwives in advanced obstetric skills (Dolo et al. 2016). Yet, like South Africa, current practice regulations in these countries are also not restructured to support midwife specialists expanded knowledge and skills (Dolo et al. 2016).

Basic midwives are registered with the SANC and are legally licensed to practice midwifery as basic midwives attending to low-risk pregnancy, delivery and postnatal care of the woman and her baby. On the other hand, midwife specialists are prepared as experts in midwifery, not just with basic midwifery knowledge and skills (Maree et al. 2018). To qualify as a midwife specialist, basic midwives must enrol for post-basic midwifery training. Upon successful completion of the training, they are deemed equipped with expert knowledge and skills to practice independently, governed by Regulation 212 (SANC 1993). Midwife specialists are trained to think critically and implement clinical competence beyond the function of a basic midwife (SANC 2020). The post-basic midwifery training enables the midwife specialists to practice as a competent and independent midwifery specialist practitioner who can provide scientific, safe and comprehensive quality midwifery care to individuals, families and communities (SANC 2020). Different from basic midwives, midwife specialist may intervene to some degree in an obstetric emergency in the absence of a medical practitioner or obstetrician (SANC 2020). The post-basic midwifery training strengthens midwives’ independent and interdependent functions to enable them to function as a clinically focussed, service orientated, independent midwives, who can render comprehensive midwifery care from the prenatal to postnatal stage (Maree et al. 2018; SANC 2020).

Midwife specialists are also prepared to act as leaders, clinical specialists, consultants, managers, researchers, change agents, advocates and educators in midwifery (Maree et al. 2018). In addition, midwife specialists are trained to give direction at local, national, regional and international levels of midwifery care (SANC 2014). These roles and responsibilities are in line with the competencies for midwife specialists as stipulated by the SANC in the competencies for midwife specialists as well as the advanced diploma in midwifery qualifications framework (SANC 2014, 2020).

Although additional competencies for midwife specialists were made available by SANC in 2014 (SANC 2014), midwife specialists in South Africa are still practising under the same scope of practice intended for basic midwives as directed by the SANC and recognised by the International Council of Nurses (ICN) (ICN 2009; SANC 2014; SANC 1990). The ICN acknowledged in 2009 that no scope of practice was available for nurses and midwife specialists globally. Moreso, the new SANC scope of practice (Regulation 2127) published in 2022 still does not include the midwife specialist; it addresses the specific roles for professional nurses, general nurses and basic midwives (SANC 2022).

Other than basic midwives being limited to care for low-risk pregnancy and deliveries only, midwife specialists are expected to manage and care for low-risk and high-risk women in pregnancy, labour and the postpartum period (SANC 2014, 2021).

International literature confirms that the lack of a scope of practice particularly for midwife specialists leaves them feeling vulnerable and excluded, resulting in them not optimally utilising their advanced knowledge and skills because of a fear of medico-legal litigation and restrictive institutional policies (Medway, Sweet & Müller 2020). The lack of a scope of practice results in midwife specialists being unable and unwilling to exercise their autonomous rights and to practice optimally as experts in the field of midwifery (Medway et al. 2020). Midwife specialists are constricted in applying their specialised acquired knowledge and skills they were deemed competent in after completion of the post-basic midwifery qualification (Butler, Fullerton & Aman 2018:169; Goemaes et al. 2020a; Hastings-Tolsma, Nolte & Temane 2021; Lesia & Roets 2013).

## Aim

The aim of the study was to explore and describe the experiences of midwife specialists in the optimal utilisation of their knowledge and skills in the public health facilities in South Africa.

## Research methods and design

### Study setting

South Africa consist of nine provinces. The study took place in public health facilities of seven provinces that offer maternal care services, where midwife specialists are rendering comprehensive midwifery care.

### Design

The research design was qualitative, explorative and descriptive in nature with a phenomenological approach to enable the researcher to fully understand the experiences of midwife specialists working in public health facilities in South Africa and the optimal utilisation of their advanced knowledge and skills. A phenomenological approach was used to gain an in-depth understanding of their lived experiences to describe the essence of their lived experiences in providing maternity care.

### Population and sampling

The population is composed of 16 midwife specialists working in the public health facilities in any of the seven provinces in South Africa. A non-probability purposive sampling strategy was used to select midwife specialists to participate in the study. The sampling method was used as it allowed the researcher to hand-pick participants currently working as midwife specialists in public health facilities in South Arica, and who were willing to share their experience in the optimal utilisation of their knowledge and skills. Participants registered as midwife specialists with SANC, working in public clinics or maternal obstetric units (MOUs) or public hospitals, with a minimum of 2 years’ working experience as a midwife specialist were included in the study. Midwife specialists with less than 2 years’ experience working at a public clinics or MOUs or public hospitals were excluded from the study.

### Data collection

Data were collected between March 2022 and June 2022. The researcher embarked on a nationwide study. Prospective participants were accessed through their nursing service managers and assistant managers of the public health facilities in seven provinces who served as gatekeepers. Research invitation letters were presented to the gatekeepers who identified potential participants in the respective midwifery units. The researcher indicated that the participants would be grouped with participants from other provinces for representation of each province in an online focus group discussion (FGD). Each focus group did not contain more than one person from the same province to ensure credibility of the gathered data. Although midwife specialists, working in public health facilities from all nine provinces in South Africa were recruited to participate in the study, only midwife specialists from seven of the nine provinces indicated their willingness to participate and were therefore included in the study. Participants indicated that FGDs during work hours would be an inconvenience, as it would interrupt continuity of care to women in need of midwifery services. The FGDs were consequently scheduled on days and at times which suited all participants.

Focus group interviews were held by the researcher to gain in-depth understanding of the experience of midwife specialists in the optimal utilisation of their knowledge and skills in public health facilities. A moderator was present in each FGD. The moderator signed a confidentiality agreement before the focus group interviews. Four focus group interviews comprising four midwife specialists in each focus group, a moderator and a researcher were held virtually using Microsoft Teams™. Microsoft Teams™ was an ideal platform for data collection in terms of affordability to cover a larger geographical area because the participants were from seven different provinces in South Africa. The option of Microsoft Teams™ was also practical for the participants in avoiding the costs of travelling from all around the country to a central location for the focus group interviews.

During the focus group interviews, all the participants were asked by the researcher to respond to and deliberate on the following central question: ‘What is your experience in optimally utilising your knowledge and skills as a midwife specialist in the public health facility where you are working in South Africa?’ Probing and other interview techniques were used to encourage depth and richness in data. Each focus group interview lasted about 1.5 h. After the transcription of the fourth focus group interview, it was determined that the same themes reoccurred, implicating data saturation.

### Data analysis

Collaizi’s seven-step descriptive phenomenological data analysis technique was followed to analyse the data, as guided by Polit and Beck (2020). The researcher had no direct or indirect personal or professional relationship with the participants other than being a midwife specialist himself (accoucheur). This was done with the aim of exploring and describing the experiences of midwife specialists working in public health facilities in South Africa, in the optimal utilisation of their knowledge and skills. Although Microsoft Teams™ has an automatic transcription tool, the researcher recorded the discussions as back up to ensure accurate transcriptions and validation of data. Participants were requested to leave their video camera on to capture their non-verbal cues as field notes. Written and verbal consent was obtained for recording of audible and visual data. The researcher also observed the moments of silence and used reflective skills to probe into participants’ responses. The focus group interviews were transcribed verbatim by the researcher following the interviews. The researcher engaged with the research data and identified themes and categories, eliciting and generating meaning. Copies of the transcriptions were sent to an independent coder who analysed the data using Collaizi’s seven steps of descriptive data analysis. A consensus discussion was held between the researcher, independent coder and supervisors to confirm the themes and categories, and the findings were contextualised using literature.

### Trustworthiness

Principles to ensure credibility, transferability, dependability and conformability were observed as measures to ensure trustworthiness by using the five principles of Lincoln and Guba (Gray & Grove 2019).

Credibility was ensured by prolonged engagement with participants to establish rapport and trust. The researcher spent sufficient time with the participants conducting small group interviews and listening to their experiences. Participants were interviewed until data saturation was reached after four focus group interviews. Member checking was carried out after data analysis and the preliminary findings of the study were shared with the participants to validate the results. Referential adequacy was ensured by recording the interviews using Microsoft Teams™ to capture the information obtained from the participants during the group interviews. Dependability was achieved by the code-recode method of analysis, where data were coded over an extended period to ensure consistency in coding. An independent coder was also employed to co-code the data independently from the researcher. Consensus on the themes and categories was reached between the researcher, independent coder and study supervisors. Transferability occurred through clear description of the research methodology. Confirmability happened by transcribing the recorded information verbatim and documenting direct quotations from the participants.

### Ethical considerations

The University of Johannesburg Research Ethics Committee and Higher Degrees Committee (REC-1279-2021; HDC-01-154-2021) granted permission to conduct the study. Further approval was obtained from the National Department of Health to include public health facilities in all the nine provinces in South Africa. In respect of right to justice, the researcher fairly selected the participants using the set inclusion criteria. The study information letter was issued to all participants beforehand to ensure that the benefits and risks involved in the participation were communicated. The letter of informed consent allowed the participants to make autonomous decision to participate in the study. Each participant gave informed, written consent to participate in the interviews and audio and visual recording of the interviews. In respect of participant’s privacy and confidentiality, the researcher generated codes specifically for discussion of data to ensure anonymity of the participants. It was made clear to the participants that confidentiality could never be ensured in groups, but appealed to the participants to keep each other’s participation and conversations confidential. Participants were allowed to withdraw from participating in the research without any penalty. All the documentation and recordings pertaining to the study were stored in a password-encrypted hard drive only accessible by the researcher.

## Results

Data were collected from 16 midwife specialists (participants) working in public health facilities, consisting of four group interviews with four participants in each group. The participants shared their experiences in the optimal utilisation of their knowledge and skills in public health facilities in South Africa. The number of years of experience indicates the participants’ experience in the implementation of their knowledge and skills. The provinces are an indication that midwife specialist from all over South Africa was included in the focus group interviews. A description of the participants’ qualifications, number of years of experience, public health care facility where they was employed, and the province is provided in Table 1.

**TABLE 1:** A description of the participant’s demography.

Focus group interview number	Participant number	Qualifications	Years of experience	Gender	Public facility	Province
Interview group 1	P1	Bachelor of nursing and midwifery	08	Female	MOU	Northwest
		Masters in post basic midwifery and neonatal nursing				
	P2	Diploma in nursing and midwifery	10	Female	Hospital	Eastern Cape
		B Cur ed et Admin				
		Masters in post basic midwifery and neonatal nursing				
	P3	Diploma in nursing and midwifery	11	Female	MOU	Gauteng
		Diploma in post basic midwifery and neonatal nursing				
	P4	Bachelor of nursing and Midwifery	07	Female	Hospital	Limpopo
		Diploma in post basic midwifery and neonatal nursing				
Interview group 2	P5	Bachelor of nursing and midwifery	09	Female	Hospital	Limpopo
		Masters in post basic midwifery and neonatal nursing				
	P6	Diploma in nursing and midwifery	11	Female	Hospital	Free State
		Diploma in Post basic midwifery and neonatal nursing				
	P7	Diploma in nursing and midwifery	08	Female	Hospital	KwaZulu-Natal
		Diploma in Post basic midwifery and neonatal nursing				
	P8	Diploma in nursing and midwifery	06	Female	MOU	Northern Cape
		B Cur ed et Admin				
		(With Post basic midwifery and neonatal nursing)				
Interview group 3	P9	Diploma in nursing and midwifery	09	Female	Clinic	Gauteng
		Diploma in Post basic midwifery and neonatal nursing				
	P10	Diploma in nursing and midwifery	07	Female	MOU	Limpopo
		B Cur ed et Admin				
		(With post basic midwifery and neonatal nursing)				
	P11	Diploma in nursing	16	Female	Clinic	Northwest
		Diploma in midwifery				
		Diploma in post basic midwifery and neonatal nursing				
	P12	Bachelor of nursing & Midwifery	13	Female	MOU	Northern Cape
		Diploma in Post basic midwifery and neonatal nursing				
Interview group 4	P13	Diploma in nursing & midwifery	09	Female	Hospital	Free State
		B Cur ed et Admin				
		(With Post basic midwifery and neonatal nursing)				
	P14	Bachelor of nursing and Midwifery	11	Female	Hospital	Northern Cape
		Diploma in Post basic midwifery and neonatal nursing				
	P15	Diploma in nursing and midwifery	12	Female	MOU	Eastern Cape
		B Cur ed et Admin				
		(With Post basic midwifery and neonatal nursing)				
	P16	Diploma in nursing	18	Female	Hospital	KwaZulu-Natal
		Diploma in midwifery				
		Diploma in Post basic midwifery and neonatal nursing				

MOU, Maternal obstetric unit.

A total of 16 female midwife specialists, four midwife specialists in each focus group, were interviewed. The average number of years of experience ranged between 6 years and 18 years. Two participants were from Gauteng, three from Limpopo, two from North West, two from Eastern Cape, two from KwaZulu-Natal, two from Free State, and three were from Northern Cape.

Three themes were identified through data analysis. Each theme with its related category is presented in Table 2.

**TABLE 2:** Summary of themes and categories.

	Theme	Category
1.	Practice with limited legal protection	1.1 Limited scope of practice for midwife specialists 1.2 Restrictions in public health facilities’ policies 1.3 Inconsistencies between education and training and practice regulations 1.4 Intense fear of litigation
2.	Fear of forfeiting knowledge and skills	2.1 Limited opportunity to utilise knowledge and skills resulting in lack of clinical ability 2.2 Inability to practice knowledge and skills
3.	Lack of trust in themselves and by other members of the multi-disciplinary team	3.1 Lack of self-recognition as an expert 3.2 Lack of recognition by line managers 3.3 Lack of recognition by physicians

Participants’ quotations are detailed next. After each quote, the focus group interview number will be indicated as ‘IG’ (Interview Group), as well as the participant number, in line with the summary of participants in Table 1.

### Theme 1: Practice with limited legal protection

The participants took part in the focus groups from March 2022 to June 2022, at the time when the revised SANC scope of practice Regulation 2127 was promulgated as Government Notice 2127 of 03 June 2022. Therefore, all participants joining the focus groups shared their experiences as practised under SANC Regulation 2598 which was proclaimed in 1984. The midwife specialists expressed that the training they received to qualify as a midwife specialist was not covered by the Scope of Practice Regulation 2598. That implies that midwife specialists receive limited protection from the previous Scope of Practice Regulation 2598. Upon reviewing the current revised Scope of Practice Regulation 2127, the conclusion can be made that the midwife specialist still receives limited legal protection should any mismanagement of patients occur.

The participants experienced that limited legal protection leave them with an intense fear of optimally utilising their knowledge and skills in the public health sector.

**Category 1.1: Limited scope of practice for midwife specialists:** Participants were aware of the extensive knowledge and skills they have following the completion of the midwifery specialist qualification one participant confirmed that:

‘As midwife specialists we are knowledgeable and skilled … that is what we studied during our training.’ (IG2, P2, F)

Participants also acknowledged that midwifery specialisation is a regulated profession, and they are obliged to adhere to the set regulations to practice. One participant mentioned:

‘Everything will need to start at SANC, our regulations are with the nursing council, they prescribe what we do and how we should do it.’ (IG4, P3, F)

However, participants experienced that they were unable to utilise their knowledge and skills optimally because there is no well-defined scope of practice for advance midwives in South Africa:

‘I feel like the use my knowledge and skills as a midwife specialist is limited as I am working in a public hospital.’ (IG1, P1, F)

‘There is no scope of practice which is the basic thing that is supposed to be guiding our practice and protecting us as midwife specialists.’ (IG4, P4, F)

**Category 1.2: Restrictions in public health facilities’ policies:** Participants were aware that midwifery practice in public healthcare facilities is regulated by the SANC. Their awareness is evidenced by their expression that the public health facilities’ policies governing their clinical practice are derived from the SANC practice regulations. An example:

‘Let’s face it, the hospital policies as she has said are going to emanate from the scope of practice itself. SANC clearly states what we are trained to do.’ (IG1, P2, F)

Participants also mentioned that SANC Scope of Practice regulations do not afford the midwife specialists autonomy to practice as experts. The policies of public health facilities seemed to restrict their practice. Participants explained that their practice was limited to basic midwifery in terms of existing SANC regulations:

‘Currently there is no scope of practice for us as midwife specialists. The one we are using is that of basic midwives R.2488, there is no mention of midwife specialist in that one but now to go into the scope of practice itself, it talks to basic midwifery practice that you are supposed to be rendering to patient.’ (IG2, P2, F)

**Category 1.3: Inconsistencies between education and training and practice regulations:** Participants were aware of what their midwifery specialisation training entailed, and the graduate attributes obtained during the midwifery specialisation training:

‘Our midwifery specialist training has prepared us for high risk and complications where advanced skills were taught and were mastered.’ (IG1, P2, F)

The continued to share that the midwifery specialisation training received was based on the midwife specialist’ competencies:

‘At the college we were trained according to the curriculum which is based on all of those competencies of the advanced midwife and were instilled in us as prescribed by the council.’ (IG2, P4, F)

Although midwifery specialist education, training and practice are regulated by the SANC, participants seemed to be aware of the inconsistencies that exist between education, training, and practice regulations as evident in the following quotations:

‘Our midwifery specialist lecturer then provided us with the competencies for a midwife specialist, but said we continue with regulation 2488. So, we know of expertise of a midwife specialist, but the scope of practice is not available.’ (IG3, P4, F)

‘So, the responsibility lies with the council to ensure that we practice all that we have learnt, by so doing SANC would have legalized our practice.’ (IG1, P2, F)

Midwifery specialist skills were also highlighted by participants. They shared that their skills were developed and deemed competent, that competency was declared during the midwifery specialist training. Yet, an inconsistency exist between education, training and practice as evidenced that were often not legally allowed in some public health facilities to exercise majority of their newly gained midwifery specialist skills, distinct in one of the participant’s quote:

‘When it comes to the hospital, you are now supposed to be adhering to the policies of the hospital and that of SANC, which are obviously so limiting to us.’ (IG1, P4, F)

Other participants shared that the rigorous training and education they received equipped them with specialised knowledge and skills to undertake more independent practising functions as midwife specialists. However, their dependent function remains. The following quotations are indicative of this:

‘I was telling you about how difficult it is to work independently here! As a midwife specialist there are no written documents to support our midwifery specialized functions.’ (IG2, P4, F)

‘I do have the knowledge and skills of managing the patients’ conditions, but then I always must wait for orders to do what I am experienced and knowledgeable to do. I must follow orders. I work under physicians.’ (IG1, P1, F)

**Category 1.4: Intense fear of litigation:** Participants reiterated they are practising with limited legal protection (R2598), and are therefore unable to execute their responsibilities as midwife specialists freely. They associated this limited legal protection with the absence of a scope of practice specifically for midwife specialists. One participant revealed:

‘R2488 is disregarding the fact that a midwife specialist has more knowledge, extensive knowledge, specialized midwifery skills and experience. So, you can’t use any of those if the scope of practice is like that.’ (IG3, P4, F)

Participants stated that the current scope of practice describes the roles and responsibilities of a basic midwife. The following quotation is an example of how most participants experienced the matter:

‘The South African Nursing Council has not yet provided us with a relevant scope of practice for midwifery specialists with specific roles and functions that I am supposed to do be performing as a specialist without that, nobody can ever know what is it that I will be able to do, or I am supposed to do….’ (IG3, P4, F)

The participants expressed that their extended knowledge and skills exceeded what is set out in the existing scope of practice. They often found themselves conflicted when dealing with obstetrical emergencies. Participants associated it with the fact that obstetrical emergencies exist, yet that is when the scope of practice is most important as their point of reference. However, it cannot be used as the current scope of practice is not relevant to midwife specialists. One of the participant shared:

‘There is a serious incongruence between what we know and what the scope of practice wants us to do, that will always land us in trouble! Because if you have done something and something went wrong, suddenly everyone remembers the scope of practice.’ (IG2, P3, F)

They proceeded to share that in cases where they were successful in their interventions during obstetrical emergencies, they were praised:

‘If you have done it and it was a success story because the patient and the neonate survived, then you are going to receive a hand of applause!.’ (IG4, P2, F)

On the other hand, unsuccessful management in obstetrical complications also existed, leaving them feeling exposed because of the absence of a scope of practice for midwife specialists. They experience such matters as a dilemma, as they are conflicted in their responsibilities, knowledge and unregulated scope:

‘People are saving themselves; people will say they don’t want to find themselves in hot waters. I know I can do this, but doing it means there is a 50% of success and 50% fail, and if I fail (God Forbids) I know I will be crucified.’ (IG4, P2, F)

‘We are afraid to lose our licenses, imagine taking risks knowing you don’t even have a legal back up?’ (IG4, P3, F)

‘We are always confronted by the situation whereby you will have to act as a midwife specialist and manage a complication even though there are no regulation in place for that. It’s a complication, patients’ lives are at stake, you must step in but, then you will find that if something is going to happen then you will be in a very huge trouble because of it! But obviously as a healthcare professional you can’t think of how you are going to be in trouble if you are prioritizing the life of a patient, you want to do your best for them.’ (IG4, P1, F)

Participants explained that the risk of litigation is unavoidable. This was associated with, for instance, when they refrained from interventions to adhere to the current scope of practice. They continued to run a risk of patient care negligence because of the specialised qualification they hold:

‘… but if you also try to work under the scope of practice and avoid the specialized interventions, then everyone is just too quick to also remind you of the specialized knowledge and skill that you are having. They will remind you of that and ask you again “Why didn’t you assist the patient”.’ (IG1, P4, F)

The same participant continued to share that as a measure to avoid legal action by SANC and the law she often resorted to defensive practice by means of clinical reports, in which she documented every minor detail about the patient:

‘As soon as the patient is admitted, even if it is the low risk, I immediately call the physician and note it in my admission report… sometimes when I deliver a woman without physician seeing them, but I still jot down that I informed the physician, you never know what will happen.’ (IG2, P4, F)

Other participants disclosed that they were often confronted by clinical situations that required them to intervene, despite the potential risk of litigations. They mentioned that in those instances they often relied on the presence of junior physicians when performing specialised interventions, as evident in the subsequent quotations:

‘With breech delivery we are the most experienced ones, because unfortunately we are in the public health sector. We are working with junior physicians most of the time … interns, who can barely manage a normal vertex, then you have no choice but to ask them to remain with you during the delivery so that at least the physician was called.’ (IG1, P2, F)

‘I will be the one leading the team to manage such a complicated delivery, but I will also be the one asking them to cover up for me, and by covering up I mean them writing notes at the end of that delivery. If I am going to be frank, I am basically saying, I am probably asking for a cover from a person who has only been in the ward for 2 weeks and I have been here for almost 12 years, because my scope of practice seemingly is limiting me. So, in a way I am practicing on the sly.’ (IG1, P2, F)

### Theme 2: Fear of forfeiting knowledge and skills

Participants communicated that they were not optimally utilising their knowledge and skills because of the lack of scope of practice for midwife specialist, as well as the restrictive public health facilities’ policies. They stated that it included the knowledge they continued to receive with continuing professional development (CPD) such as Essential Steps in Management of Obstetrical Emergencies (ESMOE) training. Therefore, the limited use of their knowledge and skills rendered them perceiving their skills and knowledge as becoming rusted.

**Category 2.1: Limited opportunity to utilise knowledge and skills resulting in lack of clinical ability:** Many participants referred to their own experiences feeling underutilised, leading to a loss in their specialised knowledge and skills:

‘As a specialized midwife you are trained to deal with high-risk clients, we are equipped with skills to manage that category of patients. So here we are unable to do that! …’ (IG4, P2, F)

‘We can also feel that we are not doing what we were taught as the college and universities truly speaking.’ (IG2, P3, F)

‘I honestly feel like being deprived to practice like this, makes one to even forget all those things, if we practice more and do this on daily basis, we become empowered more.’ (IG2, P1, F)

‘We are losing our skills. All the things we are supposed to be doing are reserved for physicians here!.’ (IG2, P2, F)

**Category 2.2: Inability to practice knowledge and skills:** Participants shared that even if they did attend CPD training such as the ESMOE, they were unable to apply their knowledge and skills because of a lack of a practice regulation and limited legal protection to practice as experts:

‘Managers are really trying to keep the skills alive, every year they organize an ESMOE training to revive our skills, but it is obviously a theoretical thing, they take both midwives and physicians.’ (IG2, P4, F)

‘The skills set is being lost, but trainings such as ESMOE are being organized for us to remain skilled and knowledgeable, but the fact of the matter is we still can’t practice because there are no protocols in place to allow us as midwife specialists to do all those life-saving skills.’ (IG4, P4, F)

### Theme 3: Lack of trust in themselves and by other members of the multi-disciplinary team

Participants shared that the restrictions to assume their specialists’ roles, because of the lack of SANC practice regulations for midwife specialists, as well as the public health facilities’ restrictive guidelines, resulted in them perceiving being less acknowledged as specialists by themselves, their line managers and physicians.

**Category 3.1: Lack of self-recognition as an expert:** Participants shared that their awareness of their extended knowledge and skills are far beyond that of a basic midwife. They reiterated that the practice regulations that exists do not differentiate their roles from that of basic midwives:

‘Our knowledge and skills exceed what is prescribed for midwives in the practice regulations, for example, we are skilled to do breech deliveries, but we cannot deliver it according to the hospital protocols.’ (IG1, P2, F)

They acknowledged that they are superior in terms of knowledge and skills; but they are perceived to be unable to provide the support to basic midwives from the specialists’ point of view. They expressed that this is detrimental to their confidence:

‘You find that when a midwife comes to you for help, we end up asking: but did you inform the physician about it? Because you would feel like a junior has done enough and there is nothing further that you can do in cases of such a patient and the next step is just to call the physician, which brings in a question again: What is the specialist midwife’s responsibility? …’ (IG1, P2, F)

Participants expressed that without recognition from SANC in terms of specific practice regulations and well-defined policies where their roles are clearly differentiated from that of basic midwives, they are unable to view themselves as specialists in the field:

‘If council can’t see us specialists in the field, we can’t even regard ourselves as specialists; we are not even practicing as such.’ (IG3, P3, F)

**Category 3.2: Lack of recognition by line managers:** Because of a lack of distinct roles and responsibilities for midwife specialists by the SANC, participants experienced that their line managers do not recognise them as specialists either:

‘Our managers are also supposed to acknowledge that we are fully skilled and knowledgeable, and it is important to ensure that we remain in practice.’ (IG2, P2, F)

Participants revealed that the disregard by their managers was evident by the delegation of responsibilities, which were often like that of basic midwives:

‘I was employed here as a midwife before I became a specialist midwife with the specialist midwife in our ward that time, but when I looked at our orientation document into the unit our documents were the same. Imagine a specialist midwife being orientated on how to do a cardiotocograph tracing! I mean this is the person who can teach everyone in the unit about the CTG.’ (IG2, P4, F)

They proceeded to share that the perceived disregard was more evident during assessments of performance when they were categorised under midwives with similar key performance area:

‘Our key performance areas in the [*Performance Management and Development System*] PMDS documents are the same as that of midwives, which means our managers also do not know how to rate us accordingly.’ (IG2, P1, F)

**Category 3.3: Lack of recognition by physicians:** Participants shared that the physicians were aware of the knowledge and skills that midwife specialists have as far as management of high-risk obstetric care and emergencies are concerned:

‘The physicians are the very ones who has trained us in theatre and in the wards as students…So yes, that the physicians knows that a midwife specialists is supposed to scrub and assist with the surgery in theatre, help them with assisted deliveries and handle other cases in the ward.’ (IG2, P2, F)

Regrettably, participants admitted that they performed below physicians’ expectations because of the restricting practice regulations and policies in the public health facilities. They mentioned that they often had to call physicians to attend to high-risk cases which they as midwife specialists were trained to safely manage. They experienced annoyance from the physicians’ because advance midwives are expected to handle certain complex cases:

‘When you admit a patient without any complication, the physician must be called. You must literally pick up a phone and call the physician and say I have admitted such and such a patient in normal labour and that sometimes frustrates physicians because the physician expects you to handle it as a specialist midwife, he needs to come in extra ordinary circumstances where the patient needs intense obstetric interventions.’ (IG2, P2, F)

The participants mentioned that the physicians often allow them to implement some of the knowledge and skills during complicated deliveries which midwives experience as a matter of convenience:

‘The other day we had a patient who came here with a very high blood pressure BP of 180/110 and the sisters were attempting to reach the physician before they could start with the patient. But the patient had an imminent eclampsia. I decided to go ahead and load the patient with magnesium sulphate and wrote a full report. I knew that possibilities are that the physician might not be impressed with this. I decided to put the patient first because I knew that it was only the matter of time before she lands into eclampsia. The physician was so happy that the patient was stabilized, but the point is I practiced my specialised midwifery skills and the physician felt like I did an extraordinary thing hence the applause! The physician was supposed to know that I am well within my rights to do what I did, in the hospital setting because of my qualified to do so.’ (IG3, P3, F)

On the contrary, participants experienced that some of the physicians does not recognise or acknowledge their skills as a midwife specialist:

‘Physicians sometimes do not acknowledge us as specialised midwives, physicians do not see us as skilled and knowledgeable professionals! It is only few of the physicians who knows that we are skilled, and they trust our judgement of the situations with patients.’ (IG3, P1, F)

## Discussion

The study explored and described the experiences of midwife specialists in optimally utilising their knowledge and skills in public health facilities in South Africa. A deep underlining tone of frustration regarding the optimal utilisation of their knowledge and skills surfaced from the participants’ experiences. Their vexations were mainly on account of the limited legal protection because of the absence of a practice regulation provided by SANC specifically for midwife specialists. Participants practised as midwife specialists under the previous regulation R.2598. Their experiences were therefore not informed by regulation R2127 of 03 June 2022. Because of the fear of litigation, participants also expressed their frustration in and the fear of forfeiting their specialised skill and knowledge. Participants similarly verbalised that they perceived themselves as midwife specialists and concurred that they have specialised knowledge and skills but were faced with doubt to label themselves as specialised midwives because of being constrained to practice as such. The lack of recognition of their specialised knowledge and skills by their line managers and physicians added to the doubt to confirm themselves as specialised midwives.

The themes highlighted that midwife specialists in South Africa practice with limited legal protection even under the current practice regulations as these regulations still do not include midwifery specialists, despite it being promulgated in June 2022. In addition, midwife specialists have a fear of becoming deprived of their specialised skills. They experience losing trust in their knowledge and skills as they do not practice it often enough. Not only do they develop a lack of confidence in their own abilities, but they perceive the multi-disciplinary team not recognising them as specialised midwives.

Participants acknowledged that the midwifery specialist training provided them with advanced knowledge and skills. Lesia and Roets (2013) confirm that midwife specialists are specialists in the field of midwifery. A midwife specialist has acquired expert knowledge and skills to include complex decision-making abilities and clinical competencies for expanded practice (Duma et al. 2012; SANC 2014). However, the lack of a scope of practice specific for midwife specialists left the participants feeling vulnerable, frustrated, unwilling and unable to perform their specialised skills because of an intense fear of litigation (Hastings-Tolsma et al. 2021; ICN 2009). In 2009, the ICN already acknowledged that there is no scope of practice specifically available for midwife specialists in South Africa and that one should be developed (ICN 2009). Although additional specific competencies for midwife specialists were made available by SANC (2014), midwife specialists are still practising under a scope of practice stating the scope of practice of basic midwives and not midwife specialists (SANC 1991, 2020). The first scope of practice was Regulation 2598 which prescribed midwives’ practices. Regulation 2598 was replaced by Regulation 2127 in June 2022. However, both Regulations 2598 and 2127 exclude the scope of midwife specialists.

The specific competencies as listed in the SANC Competencies-Midwife Specialist document (SANC 2014) preclude a statement confirming that the scope of practice and competencies of a basic midwife will be kept in mind (ICN 2009; SANC 2014). In the Advanced Diploma in Midwifery Qualification Framework, 12 competencies are relevant to basic midwives while only eight are specific to specialised midwives (SANC 2020). This translates to that 60% of the most recently recorded competencies for midwife specialists are relevant to basic midwives. Only 40% of the competencies are relevant to midwife specialists, which is not sufficient enough to completely guide midwife specialists’ clinical care.

In the research, participants shed light on standard operating procedures in public health facilities being based on the National Department of Health Guidelines for Maternity Care in South Africa (DoH 2016), which developed from the scope of practice. Therefore, midwife specialists experienced a cascading effect of not being legally protected.

Moreso, although specialised midwifery practice is regulated by SANC, participants revealed their frustration with inconsistencies among education, training and practice regulations: Even though competencies were achieved during the training to become a midwife specialist, their expert knowledge and skills were not being recognised as they were not allowed to legally carry out these skills. Specialised midwives resorted to depending on physicians in clinical management of patients, despite them obtaining advanced midwifery training. Not being able to practice independently, as expected to do after obtaining the advanced midwifery qualification, was perceived as frustrating, as the specialised midwifery training programme was specifically designed to strengthen midwives’ independent and interdependent function to render comprehensive midwifery care (Maree et al. 2018; SANC 2020). Midwife specialists are also equipped to deal with high-risk maternal cases in the absence of a physician, in the training (SANC 2020).

Considering the maternal mortality rate of 88 out of 100 000 births in facilities (Statistics SA 2022), midwife specialists play a vital role in the prevention of maternal mortality and morbidity (Goemaes et al. 2020b; Hastings-Tolsma et al. 2021; Lesia & Roets 2013). But in reality, they are not able to carry out their competencies optimally, as evident in this research.

To avoid facing litigation, midwife specialists’ recourse to defensive practice by recording intense clinical reports in which every minor detail of the delivery is recorded. Defensive recordkeeping is time-consuming and negatively impacts independent practice, teamwork and patient care (Magqadiyane 2020; Robertson & Thomson 2016). Although record keeping is essential, midwife specialists are supposed to be recognised as responsible and accountable health providers (Lesia & Roets 2013).

Contrary to the expectations that midwife specialists have extensive knowledge and skills, the participants admitted that they are gradually losing expert knowledge and skills. These findings are supported by research on clinical nursing education that found that clinical skills and knowledge are gradually lost when not practised (Faustinella & Jacobs 2018). Over time, the ability to perform a skill at a high level, or at the same level of performance you were at when you first mastered the skill, decreases if you stop practising the skill (Faustinella & Jacobs 2018; Manojkumar et al. 2022). This suggests that midwife specialists might not be able to appropriately practise or attend to obstetrical emergencies as they were trained to do (Hastings-Tolsma et al. 2021; Wibbelink & James 2015). Midwives need to have the appropriate skills to assist in reducing the maternal mortality and morbidity rate (Maree et al. 2018).

Although the participants appreciated the efforts by the Department of Health to revive their specialised midwifery knowledge and skills, their knowledge and skills remained underutilised. The participants revealed that they found ESMOE to be like the specialised midwifery training (Chabeli, Malesela & Nolte 2017) and most of the skills learnt at the ESMOE trainings were invasive and primarily for the physicians, not in the scope of practice for midwives. The participants’ reluctance in using the training they received through ESMOE was because the regulations are not explicitly mandating them to utilise their newly gained knowledge and skills through ESMOE training. This training had little value and was perceived as ‘window dressing’ by the participants.

Participants experienced inability to utilise their specialised knowledge and skills optimally, which regrettably led them to not accepting themselves as specialists. The findings highlight that the participants lost their self-confidence. The loss of self-confidence is detrimental to their clinical competence, which can in turn affect patient care (Manojkumar 2022). Yet, the maternity care guidelines, as set out by the Department of Health (DoH 2016), state that specialised midwives are first in line to receive high-risk obstetric cases and therefore require a high degree of confidence in their ability to react appropriately.

The participants continued to point out that they were not receiving recognition as specialised midwives from members of the multi-disciplinary team, including their line managers and physicians. This finding contradicts the multidisciplinary team approach for holistic management of a patient (Šanc & Prosen 2022). Although midwives provide most of the care in public health facilities (Hastings-Tolsma et al. 2021), over-reliance on physicians to handle high-risk complications occurs despite South Africa’s plans to improve maternal care practice.

### Limitations and recommendations

We are aware that this study is limited to the midwife specialists working in public facilities experiences, which might not give a detailed description of the entire specialised midwifery practice environment in South Africa. In this study, a sample size of 16 midwives was included in focus group interviews. A larger sample size of midwife specialists and including individual in-depth interviews with midwife specialist may allow for more in-depth insight into the phenomenon. However, in separate studies attached to the major study, experiences of physicians, nurse managers and medico-legal experts were explored to provide a bigger picture. The research looking into the experiences of specialised midwife in private health facilities of South Africa is already underway. The research was conducted in seven of the nine South African provinces, which limited the researcher’s intention to provide a detailed description of specialised midwifery practice in the South African context. A comparative study between South African midwife specialist and midwife specialist in other countries including the reasons for pursuing a midwifery specialisation should also be considered for future research. The midwife specialist’ optimal utilisation of their knowledge and skills and the impact of the employment site should also be further investigated.

## Conclusion

This study highlighted the lived experiences of midwife specialists in optimally using their knowledge and skills in public health facilities in South Africa. They experienced numerous and persistent barriers to utilise their specialist’s knowledge and skills, leading to midwife specialists to be discouraged and under-utilised. Midwife specialists’ experiences barriers to practice seemed insurmountable as it is directly related to a lack of practice regulations in public health care facilities. The qualitative and descriptive research design provided an in-depth description of midwife specialists’ practice environment in public health facilities. Findings suggest an urgent review of practice regulations, which includes the scope of practice from the SANC and the public health facilities’ policies. The development of a practice regulation specifically for midwife specialists in South Africa will create an enabling environment for advanced midwife specialists to assume their autonomous roles as experts. The sensitive management regarding the competencies of midwife specialists should also be considered in future.
